# Adapting the Grog survey app for alcohol screening and feedback in aboriginal and Torres Strait Islander health services: a mixed methods study protocol

**DOI:** 10.1186/s13722-025-00602-w

**Published:** 2025-09-02

**Authors:** Monika Dzidowska, James H Conigrave, Scott Wilson, Noel Hayman, Jim Cook, Lydia Gu, Darren Phung, Angela Dawson, Nikki Percival, Annalee Stearne, Marguerite Tracy, Jimmy Perry, Tanya Chikritzhs, Michelle Fitts, Teagan J Weatherall, Lynette Bullen, Craig Holloway, Kirsten Morley, Mustafa Al Ansari, KS Kylie Lee

**Affiliations:** 1 Faculty of Medicine and Health, Sydney Pharmacy School, The University of Sydney, Badham Building (A15), Sydney, NSW Australia; 2 School of Psychology and Public Health, La Trobe University, Centre for Alcohol Policy Research, NR1, Bundoora, Melbourne, 3086 Australia; 3 Aboriginal Drug and Alcohol Council (SA) Aboriginal Corporation, 155 Holbrooks Road, Adelaide, Underdale, SA 5032 Australia; 4 Southern Queensland Centre of Excellence in Aboriginal and Torres Strait Islander Primary Health Care (Inala Indigenous Health Service), 37 Wirraway Parade, Brisbane, Inala, Qld 4077 Australia; 5 School of Medicine, Griffith Health Centre (G40), Griffith University, Gold Coast campus, Gold Coast, 4222 QLD Australia; 6 School of Medicine, University of Queensland, Herston Road, Brisbane, Herston, Qld 4006 Australia; 7 Information and Communications Technology, The University of Sydney, ICT TechLab, Services Building (G12), Sydney, NSW Australia; 8 Faculty of Health, University of Technology Sydney, 235 Jones Street, Ultimo, NSW 2007 Australia; 9 Faculty of Health Sciences, Curtin University, National Drug Research Institute (NDRI), Building 609, 7 Parker Place, Bentley, WA 6102 Australia; 10 Faculty of Medicine and Health, The University of Sydney, General Practice Clinical School, Edward Ford Building (A27), Sydney, 2006 NSW Australia; 11 Menzies School of Health Research, Charles Darwin University, Mparntwe Alice Springs, Building 15, Springs Campus 10 Grevillea Drive, Alice Springs, Darwin, NT 0871 Australia; 12 NSW Health, Involuntary Drug and Alcohol Treatment Unit, Western NSW Local Health District, Bloomfield Campus, 1502 Forest Road, 2800 Orange, NSW Australia; 13 Victorian Aboriginal Health Service, 186 Nicholson Street, Melbourne, Fitzroy, Vic 3065 Australia; 14 Faculty of Medicine and Health, The University of Sydney, Central Clinical School, Lev 6, King George V Building (C39), Sydney, NSW Australia; 15 The Edith Collins Centre (Translational Research in Alcohol Drugs and Toxicology), Drug Health Services, Sydney Local Health District, Royal Prince Alfred Hospital (KGV) 83-117 Missenden Road, Camperdown, NSW 2050 Australia; 16 Burnet Institute, 85 Commercial Road, Melbourne, Vic 3004 Australia

**Keywords:** Primary care, Aboriginal and torres strait islander, First nations, Electronic screening, Alcohol, Brief interventions, Continuous quality improvement, Implementation

## Abstract

**Background:**

Routine use of brief, structured screening tools is essential to detect and provide support for Australians who drink above recommended levels. However, detecting drinking above recommended levels in Aboriginal and Torres Strait Islander Australian primary care settings is complex. Inaccuracies in completing a screening tool such as Alcohol Use Disorders Identification Test - Consumption, can lead to errors in estimating drinking in First Nations contexts where group sharing and episodic drinking make it difficult to accurately estimate alcohol consumption with tools that assume regular drinking patterns. This can lead to under-detection of drinking and a mismatch with the subsequent care that is offered. Hence, screening tools that consider these contextual factors are needed to make it easier for First Nations Australian primary care services to screen for alcohol consumption above recommended levels. Electronic screening tools offer the technical flexibility to consider the drinking contexts Furthermore, for sensitive topics such as alcohol and other drugs, computer-based screening in the general population has been shown to provide more accurate and comprehensive responses compared with face-to-face interviews.

**Aim:**

To facilitate alcohol screening and brief intervention in First Nations Australian primary care settings by adapting the Grog App – a community survey tool validated in Aboriginal and Torres Strait Islander populations for use in primary care.

**Methods:**

The project will use mixed-methods techniques across five study stages: 1 – Interest-holder consultation; 2 – technical development; 3 – re-validation and user interface acceptability; 4 – implementation in an Aboriginal and Torres Strait Islander primary care setting; 5 – acceptability study, six months after implementation.

**Discussion:**

The project will produce a novel, culturally appropriate digital health tool and implementation resources to make it easier to conduct routine alcohol screening in primary care contexts for a priority population, which may lead to increased screening and alcohol care rates. It will also provide first-ever contextual data about implementation of new health service improvement strategy focused on an electronic alcohol consumption screening tool, which is lacking in peer-reviewed literature. This study will also provide an important evidence base for using continuous quality improvement as an implementation approach in primary care settings.

## Background

Screening and brief intervention can be effective in reducing alcohol consumption in general populations [1, 2]. Yet, structural inequities make detecting drinking above recommended levels complex in First Nations Australian primary care settings [3]. First Nations clients may be less comfortable answering questions one-on-one about their alcohol use due to racism, shame, or fear of adverse consequences, such as child removal; all genuine concerns arising from the enduring consequences of colonisation [4]. It can also be uncomfortable for First Nations Australian health professionals to screen their family members or close friends, which may be considered “getting in too close” [5].

Often, individuals do not ask for help about their drinking, which may go undetected [6]. Thus routine use of brief, structured screening tools is essential to detect and provide support for First Nations Australians who drink above recommended levels [7]. Australian guidelines recommend that First Nations Australians receive screening with a validated tool ‘as part of an annual health assessment, or opportunistically’, or more frequently in high-risk groups [8]. However, uptake and use of screening tools in First Nations Australian primary care is inconsistent [9]. Screening has been shown to occur selectively such as when the client presents under the influence of alcohol or with alcohol-related injuries [6, 10]. Recent randomised trial data showed that in Aboriginal Community Controlled contexts, more than 50% of AUDIT-C screens recorded were offered as part of annual adult health checks for which clients need dedicated appointments [10]. Another analysis of the same study found that while nearly half of clients who visited the services at baseline and during implementation were not screened at all, others were screened repeatedly [11].

While routine screening is demonstrated to have benefits, it is unclear which screening tool (if any) is appropriate for clinical use as few have been validated with First Nations Australians [3]. AUDIT-C (the first three items of the Alcohol Use Disorders Identification Test) [12] is most frequently used with First Nations Australians in clinical settings. However, when screening is conducted, inaccuracies in completing AUDIT-C can result in under detection of drinking and a mismatch with the subsequent care that is offered [3, 13]. AUDIT-C typically requires the clinician to record the number of Australian ‘standard’ drinks consumed (each of 10 g ethanol). However, this requires both the client and clinician to know the volume and strength of the beverage consumed, size of a standard drink, and possess the required mathematical skills to do the calculation. Errors when completing these calculations are common [14]. Furthermore, estimating drinking can be harder in First Nations contexts where alcohol is often shared in groups and often occurs episodically [15] —making it difficult to (i) accurately track individual consumption and (ii) calculate standard drinks from non-standard drinking containers [15]. This, in turn can make it harder to answer questions on ‘usual’ quantity or frequency of consumption, such as in AUDIT-C. Hence, screening tools that consider these contextual factors are needed to make it easier for First Nations Australian primary care services to screen for alcohol consumption above recommended levels, especially when there may be no ‘usual’ drinking pattern [15]. For sensitive topics such as alcohol and other drugs, computer-based screening in the general population has been shown to provide more accurate and comprehensive responses compared with face-to-face interviews [16]. This digital tool approach has already proved successful with First Nations Australians, as ‘The Grog Survey App’ (Grog App), a population screening tool for drinking.

The Grog App is a digital survey tool, developed with First Nations Australian primary care services, drug and alcohol health professionals, community members and researchers [4]. The Grog App assesses self-reported alcohol use using: (i) a modified pictorial version of AUDIT-C [17] and a modified ‘Finnish’ method [17] that asks about the last four drinking occasions in the past 12 months; (ii) frequency of dependence symptoms (3-items derived from World Health Organization’s International Classification of Diseases 11th Revision core features) [18]; and (iii) harms from drinking. An interactive format allows individuals to select images of alcohol consumed and containers used, to indicate container fullness, and (where preferred) to describe their drinking based on what the whole group drank. The digital interface offers privacy to answer potentially sensitive questions, while also overcoming literacy barriers by delivering questions in male or female, English or Pitjantjatjara voiceover (Aboriginal language commonly spoken in parts of South Australia, Northern Territory and Western Australia) [19].

While the Grog App has been shown to be an accurate, reliable [20] and acceptable [19] tool to measure alcohol consumption among First Nations Australians in community surveys, it may have wider applications that are yet to be explored including its use as a clinical tool. The Grog App could help make screening more efficient and accurate, while also delivering tailored brief interventions and health education [19]. The Grog App could also help increase screening uptake in First Nations Australian primary care services, which have remained low despite considerable effort [21]. In this paper, we outline a protocol for testing the Grog App as a tool to improve screening for alcohol consumption in primary care.

### Aim

This study aims to facilitate alcohol screening and brief intervention by adapting the Grog App for use in primary care settings. Screening results will also be made available to the primary care clinician via clinical practice software.

## Methods

### Overall design

**Fig. 1:**
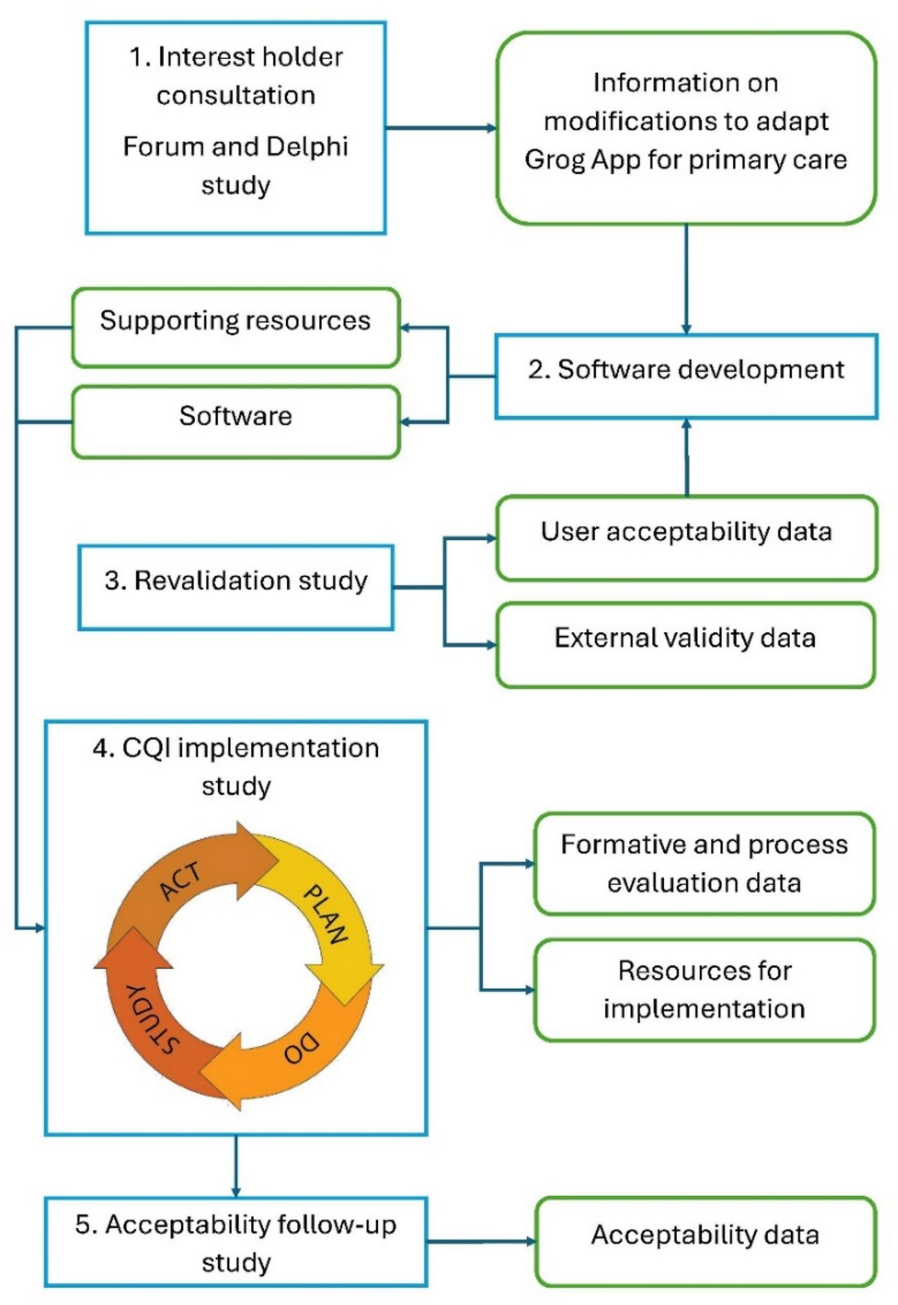
The relationship between study stages and their outcomes Blue boxes designate project stages; green boxes designate stage outcomes

The project will use mixed-methods techniques across five study stages (Fig. 1), to address the aim described above. Throughout the project, the stages will involve First Nations Australian and non-Indigenous interest holders to co-create an electronic screening tool, test technical solutions, develop implementation strategies, test acceptability for staff and clients and to disseminate the final Grog App. The project will include the following stages:

- Stage 1 – Interest holder consultation on the redesign of the original Grog App for primary care purposes via interest holder forum and a Delphi study [22].
- Stage 2 – Develop the technical solution for the modified app and connection to general practice software (‘Best Practice’).
- Stage 3 – Re-validate the app for screening sensitivity and specificity in a private primary care clinic with a high proportion of First Nations Australian clients, and test user interface acceptability.
- Stage 4 – Implement the modified Grog App at a pilot First Nations Australian primary care site using continuous quality improvement (CQI), to enable us to determine an optimal implementation strategy.
- Stage 5 – Assess acceptability of the new app in the implementation site six-months post implementation.

### Ethics, governance and community participation

This project has arisen in response to the need to adapt the Grog App, highlighted by the Southern Queensland Centre of Excellence in Aboriginal and Torres Strait Islander Primary Health Care, known as Inala Indigenous Health’ Service (NH); The project has sought and received endorsement from Marrin Weejali Aboriginal Corporation representing the study site in New South Wales (NSW), and in Queensland via Inala Community Jury for Health Research (Inala Community Jury). Subsequently, ethical approval was obtained from the Aboriginal Health and Medical Research Council of New South Wales (NSW; approval no. 2076/23), and Metro South Health, Queensland Human Research Ethics Committee (Qld, approval No. REC/2023/QMS/100707, REC/2023/QMS/100707).

**Fig. 2:**
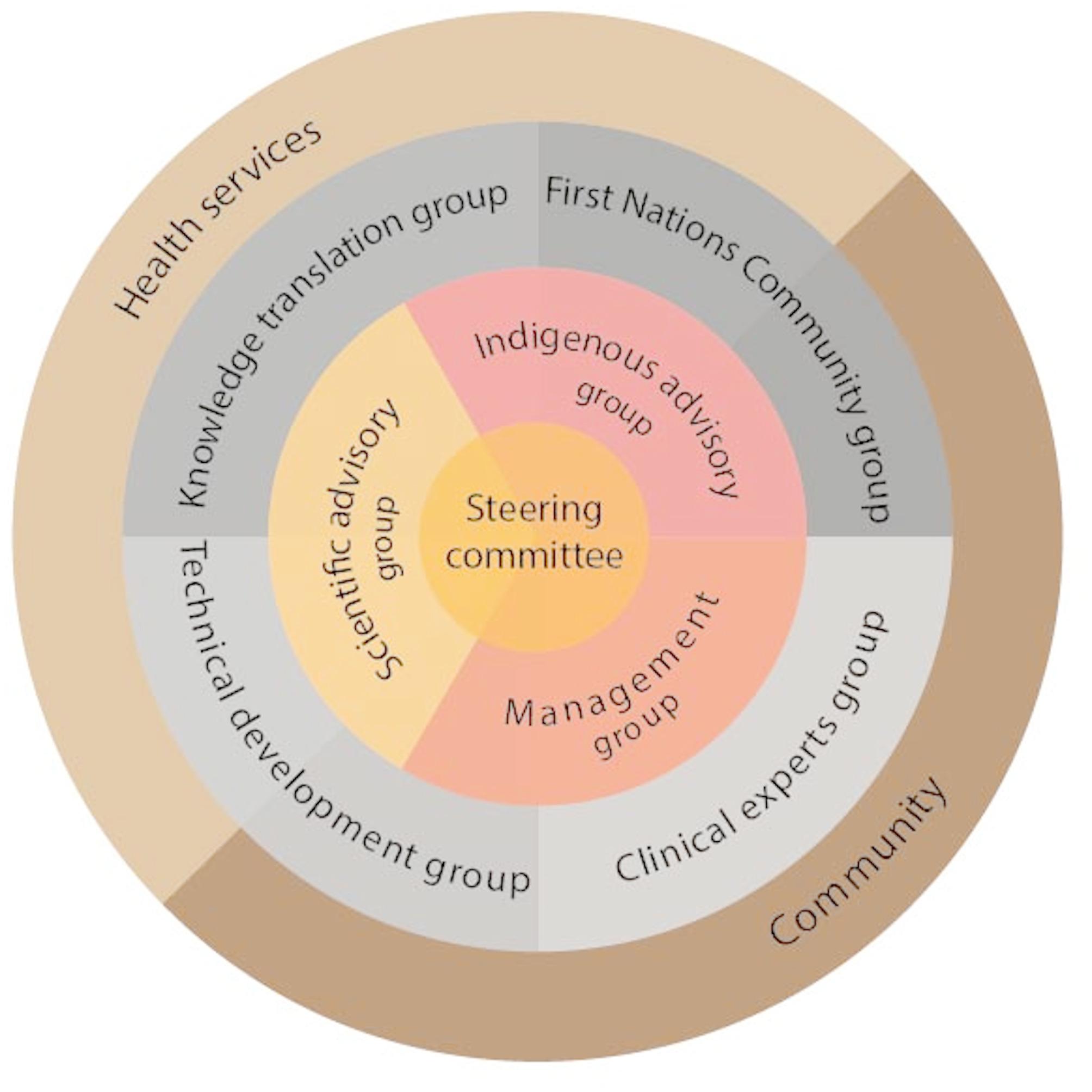
Project governance

Project governance (Fig. 2) has been designed to prioritise First Nations Australian perspectives while maintaining close collaboration, exchange of ideas and ongoing dialogue between First Nations Australian and non-Indigenous investigators and collaborators. Project governance consists of the following committees:

- A project steering committee – to oversee project delivery, comprised of a representative from each collaborating institution as well as the project management group as ex-officio members.
- An Aboriginal advisory group – to oversee project delivery to ensure cultural safety.
- Scientific advisory group – comprising all chief and associate investigators and provides academic oversight of the project.
- A project management team – responsible for day-to-day project management.

The committees are supported by expert working groups who will be engaged when their expertise is needed to inform each stage of the project. These include:

- First Nations Australian community group.
- Clinician expert group (including KC, MT, LB).
- Technical development group (including JHC, KL, JC, MT).
- Knowledge translation group (including SW, KL, NH, LB, TR).

All groups and committees comprise First Nations Australian and non-Indigenous members and are co-chaired by First Nations Australian and non-Indigenous investigators.

### Participant consent

We will provide research participants, including service staff and clients with participant information and consent forms (PICF) for every stage of the study. Participant-facing material will be written in plain English and reviewed by the communities and services where these will be implemented.

Given this research explores a sensitive topic (alcohol use), the process of recruiting health service clients and gaining their informed consent will be a multi-step process that begins at the point of engagement with potential participants. This method ensures that participants have time to consider whether they want to take part in the study even before they encounter the Grog App:

- Local health service staff with whom the clients are familiar (e.g. Aboriginal health workers, reception staff, doctor, nurse) will approach clients aged 16 or over, when they arrive at the clinic to tell them about the study and provide them with the PICF.
- Health service staff will ask clients if they have any questions and want to participate.
- If willing to participate, clients will complete the consent forms in paper-and-pen format and/or via the Grog App and be introduced to the research team member.

### Data management

A data management plan will be developed in line with the FAIR principles (findable, accessible, interoperable, and reusable) [23], the Global Indigenous Data Alliance’s (GIDA) CARE principles (Collective benefit, Authority and control, Responsibility, and Ethics) [24] and the Australian Code for the Responsible Conduct of Research 2018 [25] as guidance.

Accordingly, the participating services will co-design data collection strategies and will remain in control of data collection. They will retain joint ownership of research datasets arising from the study. Services will be consulted on drafts of all analyses and interpretation prior to dissemination of findings. Contribution on all publications will be acknowledged without identifying individual services unless they request to be named. The data management plan has been developed as part of the study protocol and includes procedures for data collection design, data aggregation, secure storage and analysis, data privacy and data governance.

### Stage 1 – interest holder consultation

This stage consists of interest holder consultation and a Delphi study. The interest holder consultation will be delivered via a two-day workshop that aims to elicit broad suggestions for adapting the original Grog App for primary care settings. Interest holder representatives from around Australia will be invited to attend with well-balanced representation of Indigenous and non-Indigenous, state/territory, as well as professional, comprising general and specialist health care professionals, community leadership, and researchers. Prior to the workshop, participants will receive information including: (i) a demonstration (demo) version of the original Grog App if they have access to an iPad tablet; (ii) questions to get forum attendees to reflect on how health professionals ask about drinking in primary care context; and how the Grog App can help with alcohol screening in this setting. Participants will have further opportunities to familiarise themselves with the existing, unmodified, Grog App during the workshop. Subsequently, a series of breakout sessions and whole group discussions will collect feedback on themes focused on modifying original Grog App features and functionality. Discussions will be recorded by scribes. The notes will be coded in NVivo and analysed to identify key themes to inform the Delphi study.

We chose to conduct a Delphi study over other techniques as it allows consensus to be built systematically and asynchronously. The Delphi study will consist of three survey rounds, with the first round introducing the ideas arising from the forum, and at least two subsequent survey rounds to resolve non-consensus.

A panel of experts will vote on how much they agree on the importance of certain app question items. The expert panel will comprise 8–12 health professionals. Eligibility criteria will be as follows: (i) aged 18+; (ii) minimum of five years of relevant health professional experience in First Nations Australian primary care, or drug and alcohol; (iii) basic computer proficiency with reliable computer and internet to access an electronic survey platform; (iv) at least 50% of panellists will identify as First Nations Australian. Recruitment will be via purposive sampling using investigator and partner networks.

Results will be descriptively analysed after each survey round. Consensus will be set at 80% agreement. Qualitative data will be coded in NVivo for themes and undergo content analysis. Aggregated de-identified visual feedback will be emailed to panellists after each round. Results from each survey round will directly inform the questions of the next survey.

### Stage 2 software adaptation and development

Based on the feedback received in Stage 1, technical work will be conducted to adapt the Grog App. This includes collaborating with the practice software developer to enable the Grog App to communicate seamlessly with their system. Technical work will be performed by the Digital Innovation TechLab team at the University of Sydney [26], with guidance from the practice software team and the project’s Technical Working Group.

A key focus of this stage will be uplifting the Grog App with FHIR (Fast Healthcare Interoperability Resources) compliant API (application programming interface) capabilities. This will ensure that the app can meet modern healthcare data standards to enable better integration with electronic medical record (EMR) systems.

The Designing for Dignity framework developed at ICT TechLab (Table 1) will guide the design process. This framework prioritises access and use of the technology developed regardless of individual functional needs and has been successfully operationalised in mental health research [27]. Our focus will be to build an inclusive web application that is based on principles of accessibility, empowerment, and usability.

An iterative approach will ensure that user feedback is continuously integrated into the design process, resulting in an application that is effective and user-friendly.

**Table 1:** Designing for dignity framework

Framework domain	Implementation principles
Prevalent use cases	Identify and analyse common user interactions and user groups to ensure that every function is intuitive and easily navigated.
Pathways to action	Create multiple ways for people with different abilities to complete the app, such as alternative input methods, guided assistance, and customisable settings.
Empowerment	Give users control over their data, with transparent access to their information, consent-based interactions, and options to manage their engagement with the application.
Inclusive design	Embed accessibility features and adaptive user flows to accommodate cognitive and physical impairments.
User-centred testing	Engage diverse user groups in iterative testing to validate that design choices align with real-world needs and lived and living experiences.

### Stage 3 – clinical revalidation of Grog app

A clinical re-validation study will be conducted at the Kildare Road Medical Centre, an urban primary care site in Western Sydney (NSW). This service provides care to up to 2000 Aboriginal and Torres Strait Islander Australian clients (aged 16+) annually. Clients who identify as Aboriginal and/or Torres Strait Islander and are 16 years of age or older will be recruited to complete the Grog App when they present at the clinic, and to talk with a First Nations Australian health professional or a general practitioner about their alcohol consumption. The comparison will be against their routine practice to screen for alcohol use. Sensitivity and specificity analyses will compare the Grog App screening responses to that of clinician screening. A minimum sample size of 188 participants is required to measure a sensitivity/specificity of 90% with acceptable accuracy [95% CI 80%, 100%; delta = 0.1]. This sample will allow us to detect statistically significant Pearson correlations as small as *r* = 0.2 which is sufficient. To provide an attrition buffer, a total sample of 200 participants will be recruited. User acceptability data will also be collected and fed back to the technical working group and developer team for any adjustments.

### Stage 4 – implementation study

Following technical development and revalidation stages, we will work with our primary care partner, Inala Indigenous Health Service to implement the Grog App into their routine practice. We will provide tablet devices and access to the App, as well as training on how to use it. We will set up local integration with Best Practice software.

We will use CQI methods to develop an optimum implementation strategy for the Inala Indigenous Health Service. The CQI approach was chosen because it allows for adaptation of the implementation strategy through iterative monitoring and formative evaluation cycles The method is flexible and responsive to a diverse and dynamic environment such as primary health care [28]. The key philosophy of CQI is that it involves everyone in the organisation in reviewing the processes of delivering care and planning and executing improvement [29, 30]. First Nations Australian primary care has seen widespread uptake of CQI since the early 2000s, with more than 200 health centres nation-wide, involved in the largest initiative - One21Seventy - between 2010 and 2012 [31]. This is likely because it is compatible with culturally sensitive approaches to First Nations healthcare including culture-centred approaches, community engagement, systems thinking that addresses the complexity of local contexts, and co-design with end-users with the aim of transferring knowledge and ensuring sustainability [32].

Inala Indigenous Health Service has an established CQI program. In the setup phase of this Stage, we will collaborate with the service to determine the best way to incorporate the implementation study into their CQI program, to minimise disruption, reduce burden, and maximise engagement. A local CQI project officer will be recruited to support the service to deliver the implementation study. A champion will also be identified from among service staff.

The implementation study will consist of three Plan-Do-Study-Act (PDSA) cycles [30], each approximately four months in duration. At the initiation of the study an introductory workshop will be held for all service staff who are interested in taking part at Inala Indigenous Health Service. The workshop will provide information about the aims of the study, and refresher CQI training. The workshop will also engage staff in the development of an implementation strategy (e.g. where will the Grog App be accessible; how will clients be introduced to the app). Inala Indigenous Health Service staff will be encouraged to take the lead on the design of the CQI strategy and its monitoring, with study staff facilitating and providing advice. Outcome measures for the initial CQI cycle will be agreed upon and will include quantitative and qualitative measures collected from staff and clients. The constructs of the RE-AIM implementation framework (Reach, Effectiveness, Adoption, Implementation, and Maintenance) will guide the design of the implementation strategy as well as the choice of outcome measures used to monitor implementation during the PDSA cycles, and the acceptability study (stage 5 below) [33, 34].

During each cycle, service staff will engage in implementing the Grog App in accordance with the agreed strategies and collect outcomes data with support from the CQI project officer and project staff. If the monitoring strategy requires it, the project management team will provide the service with regular audit-and-feedback reports in the format that best suits them. Each cycle will culminate in a workshop to review outcomes data and to adjust the implementation strategy based on these results as needed.

Each workshop will focus on the following themes to help contextualise the results and identify barriers and facilitators to implementation:

- Where are we going? (Goal)
- What is working/not working?
- Who is missing out?
- How can we do it better?
- What is feasible?
- What improvements can we make? (Ideas)
- How will we know if we are making a difference? (Measurement)

The primary outcome measure will be the proportion of Grog App completions by First Nations Australian clients aged 16 or older during each cycle (and refusals to complete the app). Depending on implementation priorities determined by baseline data, the service may choose to target a more specific group of clients. These data along with app survey responses to demographic questions will allow monitoring of patterns of self-reported alcohol consumption. Additional measures may be determined by the staff at Inala Indigenous Health Service during the CQI cycles. The project team in collaboration with Inala Indigenous Health Service staff will ensure that any additional data collection does not compromise patient or staff member privacy. Data will be descriptively analysed using R [35] and aggregated for reporting to ensure the identity of individual participants is masked and cannot be derived.

Qualitative data from service staff will be collected during subsequent workshops within each CQI cycle. The study team will collect these data by scribing the facilitated workshop sessions. Additional qualitative data may be identified by Inala Indigenous Health Service staff and appropriate ways of collection devised during the workshops. Client qualitative feedback will be collected via three feedback questions contained within the app. Additional ways of collecting feedback from patients may be suggested by staff members. Qualitative data will be coded in NVivo and undergo thematic analysis.

### Stage 5 – acceptability study

We will assess acceptability of the Grog App in primary care, six months after the implementation study is completed at Inala Indigenous Health Service.

Up to 35 participants will be sought for a one-on-one semi-structured interview (approximately 30 min in duration; up to 15 service administrators and staff, clinical and non-clinical; up to 20 clients). Method of recruitment will be co-designed with Inala Indigenous Health Service who have the connection with the community. This will optimise participation and representativeness of the sample of participants, while minimising burden on staff and clients.

Interviews will explore:

- Acceptability and feasibility of the Grog App in primary care as an alcohol screening and brief intervention tool.
- Staff experiences of client interactions based on the clients’ App results fed into Best Practice.
- Staff and client acceptability of the screening process.
- Usefulness of the tailored feedback (brief intervention).

We will use ‘yarning’ [36], an interview method, which draws on cultural protocols and prioritises the building of mutual connection and shared knowledge [36]. In ‘yarning’ the participant is encouraged to respond to a yarning topic related to the research by telling their story from their lived experience, while the researcher listens for cues. Through this method the participant is free to choose whether to disclose information [37].

An Aboriginal member of the research team with deep experience in yarning methodology will lead the interviews. To facilitate analysis, yarning interviews will be audio taped and professionally transcribed. If participants do not consent to recording, the interviewer will take notes instead. Care will be taken to de-identify transcripts. Participants will be given an opportunity to check their transcript or notes for accuracy. Qualitative data will be coded in NVivo and undergo thematic analysis.

A quantitative assessment of the Grog App’s acceptability will be conducted using data collected via the Grog App, including frequency of use, time taken to complete the App, and self-report questions asking about the App’s ease of use and acceptability.

## Discussion

### Project outcomes and outputs

The project will produce a novel and culturally appropriate digital health tool to make it easier to conduct routine screening to assess levels of drinking in primary care contexts for a priority population. The project will also result in the creation of training resources for the implementation of the Grog App in other primary care sites. Formative evaluation data obtained through the CQI process will inform the implementation of the Grog App at other primary care sites.

We expect the following overarching project outcomes:

- Embedding of a novel health technology through successful implementation of the Grog App into our pilot primary care service – Inala Indigenous Health service.
- Clinical re-validation of the Grog App following its adaptation to a different primary care setting in Western Sydney.
- Increased capacity at our implementation study site to deliver alcohol screening and brief intervention.
- Increased rates of screening and brief intervention at the implementation site.

Additional project outcomes will be developed in Stage 3 as part of the CQI-driven implementation study.

### Dissemination

The Grog App and any materials or resources arising from this project will be available to Aboriginal and Torres Strait Islander Community Controlled Health Services and other First Nations Australian community organisations. Information on the study, how to access the Grog App, scientific publications, and other resources will be disseminated through the research team’s website, gathering.edu.au. Additional dissemination methods will be co-designed with participating study sites and interest-holders.

Health services and other partner organisations taking part in the study will be regularly updated on the progress of the project in a way that suits them (e.g. newsletters, in-person presentations). For example, at the Inala Indigenous Health Service, the project team will provide regular updates to the Inala Community Jury [38], who will also approve any analysis, interpretation and publications arising from this study. We will disseminate information about the project and its outputs via our partner networks.

Research results will be disseminated through publications in peer-reviewed journals and conference presentations. Authorship will be based on intellectual contribution in accordance with the Australian Code for the Responsible Conduct of Research, 2018 [25] and the National Health and Medical Research Council’s Guidelines on Ethical Conduct in Research with Aboriginal and Torres Strait Islander Peoples and Communities [39].

### Limitations

This project is a pilot study designed to test feasibility and acceptability of a novel digital tool. The partner site involved in this study was selected because of their extensive research track record and prior involvement in helping us create the original Grog App tool (via NH, CT). Implementation in one service in an urban government-funded Queensland site may limit the generalisability of results to other primary care settings. If repeated with other partner sites, this protocol would need to be adapted based on the priorities and wishes of that partner. Nonetheless this study will provide first-ever contextual data about implementation of new health service improvement strategies focused on alcohol consumption, which is lacking in peer-reviewed literature. This study will also provide an important evidence base for future multi-centre studies.

## Data Availability

No datasets were generated or analysed during the current study.

## Abbreviations

AUDIT-C: Alcohol Use Disorders Identification Test - Consumption
CQI: Continuous Quality Improvement
EMR: Electronic Medical Record
FAIR: Findable, Accessible, Interoperable, and Reusable
GIDA: Global Indigenous Data Alliance
Grog App: The Grog Survey App
ICD-11: International Classification of Diseases 11th Revision
Inala Indigenous Health Service: Southern Queensland Centre of Excellence in Aboriginal and Torres Strait Islander Primary Health Care
NHMRC: National Health and Medical Research Council
NSW: New South Wales
PICF: Participant Information and Consent Forms
PDSA: Plan-Do-Study-Act
Qld: Queensland
WHO: World Health Organization
